# Health Literacy Mediates the Association Between Socioeconomic Status and Productive Aging Among Elderly Chinese Adults in a Newly Urbanized Community

**DOI:** 10.3389/fpubh.2021.647230

**Published:** 2021-04-09

**Authors:** Tianpei Ma, Hongdao Meng, Zhiqiu Ye, Chaoyong Jia, Min Sun, Danping Liu

**Affiliations:** ^1^Laboratory for Aging and Cancer Research, National Clinical Research Center for Geriatrics, West China Hospital, Sichuan University, Chengdu, China; ^2^Department of Health Related Social and Behavioral Science, West China School of Public Health and West China Fourth Hospital, Sichuan University, Chengdu, China; ^3^School of Aging Studies, College of Behavioral & Community Sciences, University of South Florida, Tampa, FL, United States; ^4^Department of Public Health Sciences, University of Rochester Medical Center, Rochester, NY, United States; ^5^Zhong He Community Health Service Center in Chengdu Hi-Techzone, Chengdu, China; ^6^West China School of Public Health and West China Fourth Hospital, Sichuan University, Chengdu, China

**Keywords:** health literacy, socioeconomic status, productive aging, mediation, newly urbanized community

## Abstract

Productive aging, or older adults engaging in paid or unpaid activities that produce socially valued goods or services, has been suggested to have the beneficial impact on older adults' health and well-being. We performed a cross-sectional study to examine the influence of health literacy on the relationship between socioeconomic status (SES) and productive aging among older Chinese adults in a newly urbanized community. Data was collected from 995 older adults from a newly urbanized community between June and August 2013 in Chengdu, China. We used structural equation modeling (SEM) to test the hypothesized relationship among SES, health literacy and productive aging. Results showed that education attainment and income had a direct positive effect on health literacy (β = 0.47and β = 0.15, respectively). Education had a partial indirect effect on productive aging through health literacy (β = 0.27). And health literacy was an important factor in improving the productive aging of the elderly. Interventions targeting health education and health promotion should be taken to improve health literacy of older adults under the background of urbanization, especially for those with lower SES.

## Introduction

China's older adult population is expected to increase rapidly in the next few decades, with the projected tripling of the proportion of adults aged 65 or over in the next 30 years comparing to a doubling of the same proportion in the U.S. (1). Despite improvements in living conditions and health services, older age is often associated with the decline in physical and cognitive health, as well as financial resources. There is growing concern that China's current health care and social security system will not be able to adequately meet the needs of these older residents (2). Surveys suggest that maintaining good health and having enough resources to cover health care costs are two most important concerns among older adults in China (3).

Because the great majority of older adults live in the community, encouraging older adults to participate in social activities has become a feasible way to help them stay healthy and active (56). Productive aging has been defined as any activities undertaken by an older adult, which produces socially valued goods or services, whether paid for or not (4). In general, it included employment, providing assistance to families, volunteering, and other forms of social participation (5). Researches generally showed that more participation in productive aging were associated with better health outcomes such as lower level of mortality (6), enhanced self-esteem, and improved life satisfaction (7). At the same time, productive aging would reduce the pressure of health care needs or even societal pension burden among the same population (5). However, previous studies on productive aging mainly focused on volunteering and its effect on health, resulting in a lack of understanding of other productive activities. And few studies explored the factors that affect older adults' productive aging. Moreover, these studies were conducted mostly in developed countries with well-health care systems, little is known about productive aging in China, a developing country with a vastly different cultural and social background (56).

Health literacy is an important factor that influences behavior of older adults (8). Health literacy can be defined as the degree to which individuals are able to acquire, process and understand the basic health information and services required to make appropriate health decisions (9). Also, health literacy refers to a broad range of social resources people need to access, understand, communicate, and utilize to make decisions about health (10). Older adults are a vulnerable group with regard to health literacy (8). In China, only 3.81% of people aged 65–69 had adequate health literacy in 2009 (11). A study in America demonstrated that low health literacy was a contributor to poor health outcomes and worse self-monitoring among older adults with asthma (12). Another study showed that individuals with diabetes who had low health literacy had higher odds of having unhealthy dietary habit. Although many studies on health literacy of the elderly have examined the association between health literacy and health behaviors, such as chronic disease self-management (13, 14), no study has explored the association between health literacy and productive aging among Chinese older adults.

Socioeconomic indicators such as education, income, and occupation have been found to be associated with health literacy and health behaviors (15–17). People with lower socioeconomic status (SES) are more likely to have low health literacy (18) and are less likely to engage in healthy behaviors. Furuya et al. found respondents with lower education were likely to have poorer health literacy (19). Also, Tang et al. suggested that older adults with higher SES were more actively involved in productive aging, especially regarding work and volunteering (20). It remains unclear about whether and how SES influence productive aging and the role of health literacy among Chinese older adults, as there are some qualitative evidence suggesting that Chinese older adults may have different interpretations of productive aging as compared to their western counterparts. We postulated that SES may largely determine the number of social resources that a person have access to (21), which may further influence health literacy and engagement in productive aging.

The study of the relationship between SES, health literacy and productive aging may be especially important under China's rapid urbanization process, resulting in major shifts in the population, its lifestyle and time use, from agriculture to non-agriculture. In 2011, the proportion of the urban population (51.3%) exceeded that of the rural population for the first time in history (22). The urbanization process displaced many formerly non-urban residents and created many new urbanized communities. The resulting changes in population age composition is further exacerbated by increased mobility of young people especially from the rural areas to cities and broke the traditional model of family support. Consequently, the burden of old-age care had been shifted from family to the state or other social organizations (23). The impact of urbanization on health is two-fold. On the one hand, changes in living environment and lifestyle are all related to the rapid growth of urbanization (24). Many factors, such as deteriorating air quality, increased high-calorie intake, and reduced social interaction with neighbors, affect the health of the elderly. On the other hand, people tend to have better access to quality health services and other community resources, including health information (25). Thus, health literacy may change as health information becomes more accessible (11).

Most previous researches focused on the traditional urban elderly or the rural elderly, while there are few studies researched on productive aging among the new urbanization residents. This study examined the effects of SES and health literacy on productive aging among elderly in a newly urbanized community in China and had important implications for health promotion in older adults.

**Figure 1:**
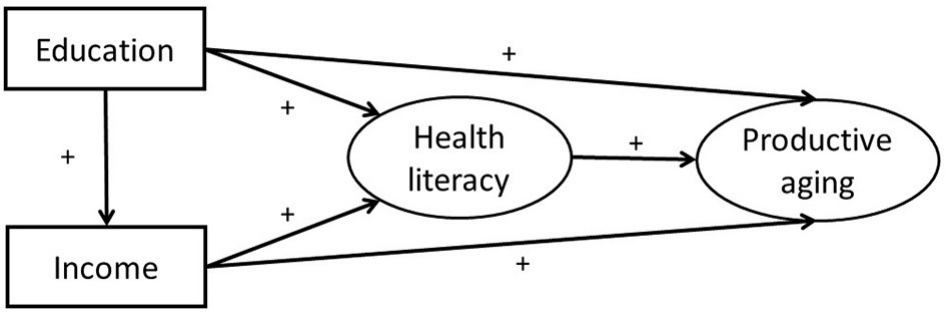
The theoretical model and hypotheses.

Drawn from the extant literature and empirical support, we tried to establish the relationships among SES, health literacy and productive aging. We hypothesized that health literacy would mediate the effect of socioeconomic on productive aging. The theoretical hypotheses and the mediator model were shown in Table 1 and Figure 1. We assumed that health literacy directly affected productive aging. Education and income affected productive aging both directly and indirectly. This study would further explore the intermediary effect of health literacy on the relationship between education, income and productive aging.

**Table 1:** The theoretical hypotheses.

**Hypotheses**
1. Education has a positive effect on income.
2. Education has a positive effect on health literacy.
3. Income has a positive effect on health literacy.
4. Health literacy has a positive effect on productive aging.
5. Education has a direct positive effect on productive aging.
6. Income has a direct positive effect on productive aging.
7. Education has an indirect positive effect on productive aging through the mediating effect of health literacy.
8. Income has an indirect positive effect on productive aging through the mediating effect of health literacy.
9. Education has an indirect positive effect on health literacy through the mediating effect of income.

## Methods

### Participants

The cross-sectional study was conducted between June and August 2013. The face-to-face questionnaire survey was conducted in a newly urbanized district in the suburbs of Chengdu, Sichuan Province. The sample inclusion criteria were that the participants were 65 years old and above and had resided continuously in the community for more than 6 months.

We calculated the sample size using the following formula: $n=\left(\frac{Z_{\alpha /2}}{\delta }\right)\wedge 2\times \pi \times \left(1-\pi \right)$ (26), =9.94% (which was the overall health literacy rate of urban residents in the first Chinese Residents' Health Literacy Survey in 2009) (27) = 2%, π = 0.05, =1.96. Based on this formula, the sample size was calculated as 818. Considering possible dropout, we increased the sample size by 20%, which resulted in a final sample size of 1,000.

Participants were recruited through simple random sampling methods. The SAS 9.3 proc plan program was used to randomly select respondents from a database of elderly adults established by the Community Health Service Centers. A total of 1,000 older residents were surveyed face-to-face by trained investigators; of these, we obtained and analyzed 995 (99.5%) valid responses. The study protocol was approved by the Institutional Review Board of West China School of Public Health, Sichuan University. Informed consent was obtained from each participant following a detail explanation about the purpose of the study.

### Measures

The questionnaire included four parts, (a) socioeconomic status, (b) other demographic characteristics, (c) health literacy, (d) productive aging.

In this study, we used education and personal monthly income to measure SES of participants. Education was defined as four different levels (1 = no formal education, 2 = primary school, 3 = junior school, 4 = high school and above). Personal monthly income was categorized into four levels, ranging from <1,000¥ to more than 3,000¥ by an increment of 1,000¥.

Other demographic information mainly includes gender, age, marital status and living arrangements. Age was categorized as 65~, 70~, 75~ and 80~ years. Marital status was measured as single, married, divorced, and widowed. Living arrangements included four types: living with spouse, living with children, living with spouse and children and living alone.

We adapted the Chinese Residents' Health Literacy Questionnaire to measure literacy. This questionnaire was originally designed by the National Health and Family Planning Commission of the People's Republic of China (28). We adapted the questionnaire by selecting items closely related to the health literacy of the elderly and adding items about health knowledge of the prevention of common chronic disease per expert opinions. The revised questionnaire of health literacy (see Appendix 1) had 29 items, divided into three domains, including knowledge and belief (*n* = 19), behavior (*n* = 8) and skills (*n* = 2). Each item was given a score of 1 point if a correct response was provided. The total score ranges from 0 to 29, with the higher score indicating a higher level of health literacy. Participates receiving a score of 23 [which was 80% of the full marks of 29 (29)] or higher were categorized as having adequate health literacy. This scale has been shown to have good psychometric property (Cronbach' alpha 0.886) (55).

Productive aging describes productive activities taken by the elderly. In our study, five productive activities were examined: employment, doing household chores, caregiving, volunteering and learning. For employment, we asked participants whether they maintained work after retirement (0 = no, 1 = yes), including both full-time and part-time work. For the other four items, participants were asked to indicate the frequency of each activity (see Appendix 1). This four items response ranged from 0 = never to 3 = frequently. The total score ranged from 0 to13, with a higher score indicating a higher level of productive aging. This scale has a Cronbach's α coefficient of 0.731.

### Statistical Analysis Method

Descriptive statistics were used to examine the distribution of each variable. Pearson coefficient was used to analyze the correlation of major constructs of the theoretical model. IBM SPSS 20.0 software was used for descriptive statistical analysis and correlation analysis. Structural equation model (SEM) was used to further test the hypothesized relationship between the four dimensions, education, income, health literacy and productive aging. IBM SPSS AMOS 24.0 software was used for establishing structural equation model. A *P*-value < 0.05 was defined as statistically significant. The structural equation model used bootstrap maximum likelihood estimation. Model fit was assessed through several commonly reported fit statistics, such as RMSEA (root mean square error of approximation) <0.08, NFI (normal fit index), CFI (comparative fix index), IFI (incremental fit index) of 0.90 or above, PCFI (parsimony comparative fix index), PNFI (parsimony normed fit index) of 0.50 or above (7).

## Results

### Characteristics of the Participants

This descriptive information of 995 participants was provided in Table 2. The average age of respondents was 71.8 years (SD = 13.9) and approximately half were women (52.6%). A majority of the participants reported receiving a primary school (45.5%) or no formal education (31.1%). Approximately three-fourths were married (75.4%). Less than half of the elderly reported living with spouse and children (43.7%) and the vast majority of the residents earned <2,000 RMB a month (80.2%). The mean score of health literacy for the elderly was 12.6 (SD = 6.0). Only 8.5% of the participants were above 23, the threshold of adequate health literacy. The mean score of knowledge and belief literacy, behavior literacy and skill literacy were 7.4 (SD = 4.6), 4.0 (SD = 1.6) and 1.2 (SD = 0.6), respectively. Additionally, the average score of productive aging for the elderly in this survey was 6.0 (SD = 2.7). And 2.0% of samples answered No to all questions. You may insert up to 5 heading levels into your manuscript as can be seen in “Styles” tab of this template. These formatting styles are meant as a guide, as long as the heading levels are clear, Frontiers style will be applied during typesetting.

**Table 2:** The characteristics of the participants (*n* =995).

	**Frequency (*n*) or Mean**	Percentage (%) or SD^*^
Gender		
Male	472	47.4
Female	523	52.6
**Age(years)**		
65~	431	43.3
70~	282	28.3
75~	180	18.1
80+	102	10.3
**Married status**		
Single	2	0.2
Married	750	75.4
Divorced	7	0.7
Widowed	236	23.7
**Living situation**		
With spouse	284	28.5
With children	229	23.0
With spouse and children	435	43.7
Live alone	47	4.7
**Education**		
No formal education	309	31.1
Primary school	453	45.5
Junior school	143	14.4
High school and above	90	9.0
**Personal monthly income (RMB)**		
<1,000	62	6.2
1,000~1,999	736	74.0
2,000~2,999	142	14.3
3,000+	55	5.5
Total score of health literacy	12.6	6.0
Knowledge and belief literacy	7.4	4.6
Behavior literacy	4.0	1.6
Skill literacy	1.2	0.6
Total score of the productive aging	6.0	2.7

* *SD, standard deviation*.

### Productive Aging Status of the Participants

Table 3 shows productive status of the elderly. Only 4.2% of older adults reported participating in work. The elderly who often did household chores and cared for their families accounted for 59.0% and 46.0%, respectively. A small proportion of the participants frequently participated in volunteer and learning activities, taking up 3.8 and 17.1%, respectively.

**Table 3:** Productive aging status of the participants.

	**Paid work**		**Doing household chores**		**Caregiving**		**Volunteering**		**Learning**	
	***n***	**%**	***n***	**%**	***n***	**%**	***n***	**%**	***n***	**%**
No	953	95.8								
Yes	42	4.2								
Never			97	9.7	222	22.3	764	76.8	167	16.8
Rarely			115	11.6	114	11.5	53	5.3	532	53.5
Occasionally			196	19.7	201	20.2	140	14.1	126	12.7
Frequently			587	59.0	458	46.0	38	3.8	170	17.1

### Correlation Among SES, Health Literacy and Productive Aging

As presented in Table 4, the correlations between pairs of education, personal monthly income, health literacy and productive aging were statistically significant (*P* <0.01). Productive aging was positively correlated with health literacy, education and personal monthly income. Participants who had higher health literacy scores had higher education and income level. Moreover, education was positively correlated with personal monthly income.

**Table 4:** Correlation among SES, health literacy and productive aging.

		**1**	**2**	**3**	**4**
1	Education	1			
2	Personal monthly income	0.395^**^	1		
3	Health literacy	0.476^**^	0.301^**^	1	
4	Productive aging	0.357^**^	0.184^**^	0.413^**^	1

** *P <0.01, two-tailed*.

### The Mediating Effect of Health Literacy on the Association Between SES and Productive Aging

**Figure 2:**
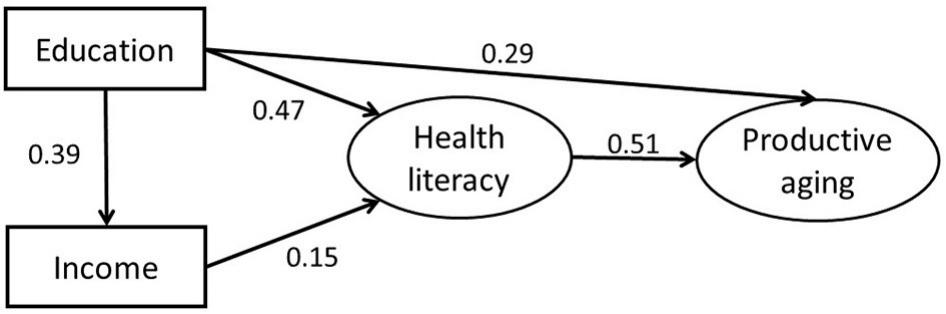
The final model and standardized model path coefficient.

This study established a structural equation model, shown in Figure 1, to examine how productive aging would be influenced by income, education and health literacy. The model fit indices of the hypothesized model were RMSEA = 0.108, NFI = 0.820, CFI = 0.831, IFI = 0.832. Those fit indices of the hypothetical model failed to meet the fitness criteria. Also, income had no significant direct effect on productive aging (β = 0.00, *P* = 0.914). Thus, this model needed to be modified. After adjusting, the final output model was shown in Figure 2, which presented the standardization path coefficient. With addition of other socio-demographics (gender, age, married status and living situation) as covariates, the arrow direction among the core variables in the model remained unchanged, in addition, the corresponding coefficients did not change significantly. Therefore, the socio-demographics were not confounding factors. Specifically, the absolute fit index, the incremental fix index and the parsimonious fit index of final model all met the fitness criteria (RMSEA = 0.058, NFI = 0.937, CFI = 0.951, IFI = 0.951, PCFI = 0.655, PNFI = 0.646).

Bias-corrected bootstrap with 2,000 replications using maximum likelihood estimation was employed for each path. The results of the estimation are shown in Table 5. If the 95% confidence interval (95% CI) of the estimation of the path coefficient does not include 0, it means that the direct, indirect, or total effect are statistically significant. Education had a positive effect on income (β = 0.39, *P* < 0.001). Education exerted a positive influence on health literacy (β = 0.47, *P* < 0.001). Income exercised a positive impact on health literacy (β = 0.15, *P* < 0.001). Health literacy had a direct positive effect on productive aging (β = 0.51, *P* < 0.001). Education had a direct positive effect on productive aging (β = 0.29, *P* < 0.001). Education influenced health literacy mostly directly. The proportion through the impact of income was very small. Finally, the final results supported all hypotheses except hypothesis 6.

**Table 5:** Significance test of the mediating test.

**Model pathways**	**Estimated**	**95% CI**
**Total effects**		
Income ← Education	0.395	0.332–0.454
Health literacy ← Education	0.533	0.472–0.595
Health literacy ← Income	0.152	0.086–0.217
Productive aging ← Health literacy	0.508	0.407–0.626
Productive aging ← Education	0.560	0.484–0.632
Productive aging ← Income	0.077	0.044–0.116
**Direct effects**		
Income ← Education	0.394	0.332–0.454
Health literacy ← Education	0.474	0.405–0.543
Health literacy ← Income	0.152	0.086–0.217
Productive aging ← Health literacy	0.508	0.407–0.626
Productive aging ← Education	0.289	0.186–0.383
**Indirect effects**		
Productive aging ← Education	0.271	0.209–0.349
Productive aging ← Income	0.077	0.044–0.116
Health literacy ← Education	0.060	0.034–0.089

Table 6 displays the significance test of three mediating pathways of final model. The results showed the 95% CI of the estimates of the three-mediation path did not include 0, indicating that mediating effects of health literacy on the influences of education and income on productive aging were both statistically significant (30). With respect to the influence of education on productive aging, we found that the total effect, direct effect and indirect effect of this path were all statistically significant, suggesting that education influenced productive aging both independently and partially through health literacy. However, the effects of income on productive aging was completely mediated through health literacy. We also found that education influenced health literacy partially through the effects of income.

**Table 6:** Significance test of every mediating pathway.

**Model pathways**	**95% CI**
Health literacy ← Income ← Education	0.134–0.403
Productive aging ← Health literacy ← Education	0.004–0.013
Productive aging ← Health literacy ← Income	0.002–0.007

## Discussion

This study is dedicated to explore how SES (education, income) and health literacy influence productive aging among older Chinese adults in a newly urbanized community for the first time, thereby providing theoretical support for promoting health of the elderly.

The research showed that the mean score of productive aging for the elderly in this survey was only 6.0 (SD = 2.7). It suggests that the level of productive aging was generally low, especially with respect to the engagement in work, volunteer and leaning activities. The productive aging of elderly residents was mainly focused on family contribution, such as doing household chores (59.0%) and caregiving (46.0%). A similar situation of productive aging has been reported by Li et al. that older Chinese adults had a low participation rate in paid employment but high participation in assisting family. In newly urbanized communities, most elderly lost their land and can no longer do farm work. They may spend more time doing housework and caring for grandchildren, which is a common phenomenon in China, a family-oriented, collectivist country (31, 32). At the same time, the relevant policies and infrastructure are still not perfect in the newly urbanized communities, as well as jobs and volunteer service organizations are scarce for the elderly (33). Accordingly, older adults rarely have the opportunity to continue working or volunteering, let alone studying. Older adults who engaged in more hours of volunteering or lifelong learning reported higher levels of well-being (34, 35). Therefore, the government should encourage the elderly to participate in social activities and make lifelong learning by creating an environment supporting productive aging.

In addition, the mean score of health literacy for the elderly was 12.6 (SD = 6.0). Only 8.5% of the participants had adequate health literacy. It meant that health literacy of the participants was rather low, lower than the results of urban residents reported by the first *Chinese Residents' Health Literacy Survey* in 2009 (9.9%) (27). This reflected the lack of health related knowledge, behavior and skill among the elderly in the new urbanized community. 76.8% of participants had a primary school level of education or below in this study. Despite a lot of health information on the web, the majority of older adults cannot use the internet (36).

The model demonstrated that health literacy positively influenced productive aging. Considerable previous researches found that health literacy could affect the behaviors of the elderly (37–39). Those with higher health literacy may better adopt positive aging to promote their health (40). A prior study revealed health literacy increased utilization of preventive care among older adults in Taiwan (13). Peterson et al. verified that patients with higher health literacy reported less barriers to complete cancer screening (41). In the current research, we found that health literacy would directly positively influence productive aging. As a collection of various active behaviors in the life of the elderly, productive aging such as volunteering and working were generally related to good health outcomes (42, 43). The elderly with higher health literacy may have more health information and more likely to take productive aging to promote their physical and mental health in the urbanized community.

The model also showed higher SES positively influenced health literacy, which was basically consistent with previous studies (44, 45). On the one hand, high education level was closely related to better health literacy. It may be that education affects older adult's access to information and resources and also willingness to obtain information (46). People with low education maybe not understand and use medical information in their daily life. On the other hand, the higher the income, the better the health literacy. This was consistent with the results of a survey conducted by Su et al. in Korean older adults (47). It may be because older people with higher incomes have more social resources and social support to improve their health literacy. At the same time, education also affected health literacy through income. Therefore, SES was important influencing factors of health literacy.

Our study found that education had a positive direct effect on productive aging, which was similar with previous studies. Horowitz et al. revealed education was an important way to promote productive aging (48). Other studies found that older adults with a lower education level were less likely to participate in social activities (49, 50). Policies that aims to promote the productive participation of older adults in social activities should focus on groups with lower education levels (51).

The important finding of this study was that education and income influenced productive aging through health literacy. This indicates that health literacy mediates the relationship between socioeconomic status and productive aging among the elderly in the newly urbanized community. Considering that the SES of the elderly is difficult to change, health literacy is a key factor in improving the productive aging of the elderly in urbanized communities. The level of productive aging could be increased through the improvement of health literacy (52). We can take health education and health promotion strategies for the elderly, such as conducting live health lectures or improving the accessibility and readability of health information, to facilitate the understanding of health-related information, and hence improve health literacy (53, 54).

The main limitation of this study is that it is a cross-sectional survey, it does not validate the causal relationship between SES, health literacy and productive aging. Our results only provide information concerning the direct and indirect influencing factors of productive aging among these older adults in the context of urbanization.

## Conclusions

In conclusion, this study reveals that SES and health literacy are significantly related to productive aging among older adults in the newly urbanized community. Education and income have direct positive effects on health literacy. Health literacy has a direct positive effect on productive aging. Education also has a direct positive impact on productive aging, while the direct positive impact of income on the productive aging has not been affirmed. Health literacy mediates the influence of SES on productive aging. Thence, interventions targeting health literacy should be prioritized for older adults to promote productive aging under the background of urbanization, especially for those with lower SES.

## Data Availability Statement

The raw data supporting the conclusions of this article will be made available by the authors, without undue reservation.

## Ethics Statement

The studies involving human participants were reviewed and approved by Institutional Review Board of West China School of Public Health, Sichuan University. The patients/participants provided their written informed consent to participate in this study.

## Author Contributions

TM and DL conceptualized the idea. DL, CJ, and MS collected the data. TM performed the statistical analyses and wrote the first draft of the manuscript. DL, HM, and ZY critically revised the manuscript. All the authors checked and approved the final manuscript.

## Conflict of Interest

The authors declare that the research was conducted in the absence of any commercial or financial relationships that could be construed as a potential conflict of interest.
