# Supplementary material for: Health Literacy Mediates the Association Between Socioeconomic Status and Productive Aging Among Elderly Chinese Adults in a Newly Urbanized Community
Source: Front Public Health. 2021 Apr 9;9:647230. doi: 10.3389/fpubh.2021.647230 (PMC8062760; doi:10.3389/fpubh.2021.647230)
Supplement: Supplementary file 1 [file Data_Sheet_1.docx]

Supplementary Material

# Questionnaire

**Part 1. Demographic information**

1. Gender: 1) Male 2) Female

2. Age:

3. Married or not?

1) Yes 2) No (never married, divorced or widowed)

4. Who do you currently live with?

1) With spouse 2) With children

3) With spouse and children 4) Living alone

5. Education level:

1) No formal education 2) Primary school

3) Junior school 4) High school and above

6. What is your average monthly income?

1) <1000 yuan 2) 1000～1999 yuan 3) 2000～2999 yuan 4) 3000+ yuan

**Part 2. Health literacy**

1. Choose the following statement you agreement.

1.1 Health is no physical illness.

1) Agreement 2) Disagreement 3)Not sure

1.2 People who are in good health do not need to pay attention to health problems.

1) Agreement 2) Disagreement 3)Not sure

1.3 The fatter the body, the better the body.

1) Agreement 2) Disagreement 3)Not sure

2. Injection is better than taking medicine. If you are sick, you should injection.

1) Agreement 2) Disagreement 3)Not sure

3. The psychological problem is not sick, no need to see a doctor.

1) Agreement 2) Disagreement 3)Not sure

4. Rapid weight loss is one of the early signs of cancer.

1) Agreement 2) Disagreement 3)Not sure

5. Washing your hands helps prevent influenza.

1) Agreement 2) Disagreement 3)Not sure

6. Which of the following is the normal range of temperature for an adult's armpits?

1) 35 to 36℃ 2) 36 to 37℃ 3) 37 to 38℃ 4) Not sure

7. Can you use a thermometer and read the temperature value measured by the thermometer?

1) Yes 2) No

8. Which of the following do you think a healthy lifestyle? (multiple choices)

1) Smoking cessation, alcohol restriction 2) Balanced diet

3) Getting along with people 4) Controlling weight

5) Eat more health supplements and nutrients

6) Eat more and sleep more 7) Exercise regularly 8) Not sure

9. Which of the following diseases do you think passive smoking causes? (multiple choices)

1) Lung cancer 2) Coronary heart disease

3) Chronic obstructive pulmonary disease 4) Caries 5) Cataract

6) Affecting fetal development 7) Not sure

10. Which of the following data do you think is the normal blood pressure range?

1) 140/95 mm Hg 2) 120/80 mm Hg 3) 150/100 mm Hg 4) Not sure

11. Which of the following conditions do you know are prone to high blood pressure? (multiple choices)

1) Obesity 2) Eat more salt 3) Mental stress 4) Smoking

5) Lack of exercise 6) Excessive drinking 7) Eating more sugar 8) Not sure

12. In order to prevent high blood pressure, how many grams of salt do you usually eat per day for adults?

1) 2 grams 2) 6 grams 3) 9 grams 4) 12 grams 5) Not sure

13. Do you know which of the following symptoms of diabetes? (multiple choices)

1) Eat more 2) Drink more 3) Mouth much more 4) Lose weight

5) Eat more sweets 6) Not sure

14. Which of the following practices are good for preventing diabetes? (multiple choices)

1) Controlling weight 2) Scientific diet 3) Maintaining a happy mood

4) Moderate exercise 5) Learning preventive health knowledge 6) Not sure

15. Which of the following practices are beneficial for preventing the development of senile chronic bronchitis? (multiple choices)

1) Keep warm and prevent colds

2) Reduce the inhalation of harmful gases and particles

3) Quit smoking 4) Strengthen exercise and improve resistance 5) Not sure

16. Which of the following practices are beneficial for preventing the development of chronic obstructive pulmonary disease? (multiple choices)

1) Quit smoking 2) Reduce the inhalation of harmful gases and particles

3) Protect from cold and prevent colds

4) Strengthen physical exercise and improve immunity 5) Not sure

17. Which of the following do you think are the causes of chronic gastroenteritis? (multiple choices)

1) Insufficient chewing 2) Eating irritating food 3) Excessive smoking

4) Drinking strong tea, alcohol, and coffee for a long time

5) Long-term use of multiple drugs 6) Not sure

18. What should be noted in the daily life of patients with bone hyperplasia? (multiple choices)

1) Avoid long-term strenuous exercise 2) Exercise properly

3) Treat injury in time 4) Lose weight 5) Not sure

19. Which of the following practices are good for preventing rheumatoid arthritis? (multiple choices)

1) Strengthen exercise, keep fit 2) Avoid wet environment

3) Pay attention to work and rest 4) Maintain a good attitude

5) Pay attention to prevent and control infection 6) Not sure

20. The following statements about antibiotics, what you think are correct:

1) The cold must be eaten immediately antibiotics.

2) Antibiotics can be purchased according to the condition.

3) Antibiotics should be used under the guidance of a doctor.

4) Antibiotics can kill bacteria and kill viruses.

5) Not sure

21. Which is the best practice for coughing for more than two weeks, or hemoptysis, hypothermia, fatigue, night sweats, etc?

1) First squat, see the doctor when you feel bad

2) Buy some cold medicine yourself

3) Go to the hospital immediately to see a doctor

4) Not sure

22. Which of the following foods can be used to supplement calcium? (multiple choices)

1) Dairy and dairy products (milk, goat's milk and milk powder, etc.)

2) Beans and soy products (soya, tofu, etc.)

3) Seafood (crab, seaweed, etc.)

4) Meat and eggs (mutton, eggs, etc.)

5) Vegetables (black fungus, mushrooms, etc.)

6) Fruits and dried nuts (apples, peanuts, etc.)

7) Not sure

23. What you think is correct?

1) Vegetables and raw meat should be used with different chopping boards for cooked and cold dishes.

2) Knife cutting the vegetable, can be used to cut cooked meat after water it.

3) Chopping board cutting the raw meat, can be used to cut cooked meat after water it.

4) Not sure

24. What the following statements about the shelf life of foods are correct?

1) Foods that have passed the shelf life can be eaten as long as they don't look bad.

2) Do not eat foods that exceed the shelf life.

3) After the food that has passed the shelf life, it can be eaten after cooking.

4) Not sure.

25. If you are scratched or bitten by a dog or cat, you will:

1) Inject rabies vaccine immediately

2) Wash the wound immediately with soap and water and inject rabies vaccine as soon as possible 3) Wash with water

4) Treat wounds with folk remedies 5) Not sure

26. What diseases can be caused by drinking contaminated water? (multiple choices)

1) Hepatitis A 2) Hepatitis B 3) Poisoning 4) Cancer

5) Dysentery 6) Measles 7) Not sure

27. If someone finds gas poisoning, you will: (multiple choices)

1) Open the window

2) Move the gas poisoner to the fresh, well-ventilated place as soon as possible

3) Call the emergency number

4) Lift the gas poisoning person to a cool place and give him some vinegar

5) Not sure

28. When you need emergency medical assistance, you should call:

1) 120 2) 119 3) 122 4) Not sure

29. Have you had a health checkup every year for the past 2 years?

1) Yes 2) No

**Part 3. The productive aging status**

1. Do you currently work?

0) No 1) Yes

2. Do you do housework?

0) Never 1) Sometimes 2) Often 3) Usually

3. Do you care for other members of the family (such as spouses, children, grandchildren)?

0) Never 1) Sometimes 2) Often 3) Usually

4. Have you participated in community volunteering or social welfare activities?

0) Never 1) Sometimes 2) Often 3) Usually

5. Do you currently study?

0) Never 1) Sometimes 2) Often 3) Usually
